# Food-Related Impulsivity in Obesity and Binge Eating Disorder—A Systematic Update of the Evidence

**DOI:** 10.3390/nu9111170

**Published:** 2017-10-27

**Authors:** Katrin E. Giel, Martin Teufel, Florian Junne, Stephan Zipfel, Kathrin Schag

**Affiliations:** 1 Department of Psychosomatic Medicine and Psychotherapy, Medical University Hospital Tübingen, 72076 Tübingen, Germany; florian.junne@med.uni-tuebingen.de (F.J.); stephan.zipfel@med.uni-tuebingen.de (S.Z.); kathrin.schag@med.uni-tuebingen.de (K.S.); 2 Department of Psychosomatic Medicine and Psychotherapy, University of Duisburg-Essen, LVR-Hospital, 45147 Essen, Germany; martin.teufel@uni-due.de

**Keywords:** binge eating disorder, eating disorders, food, impulsivity, inhibition, obesity, reward

## Abstract

The specific eating pattern of Binge Eating Disorder (BED) patients has provoked the assumption that BED might represent a phenotype within the obesity spectrum that is characterized by increased impulsivity. Following the guidelines of the PRISMA statement (preferred reporting items for systematic reviews and meta-analyses), we here provide a systematic update on the evidence on food-related impulsivity in obese individuals, with and without BED, as well as normal-weight individuals. We separately analyzed potential group differences in the impulsivity components of reward sensitivity and rash-spontaneous behavior. Our search resulted in twenty experimental studies with high methodological quality. The synthesis of the latest evidence consolidates conclusions drawn in our initial systematic review that BED represents a distinct phenotype within the obesity spectrum that is characterized by increased impulsivity. Rash-spontaneous behavior in general, and specifically towards food, is increased in BED, while food-specific reward sensitivity is also increased in obese individuals without BED, but potentially to a lesser degree. A major next step for research entails the investigation of sub-domains and temporal components of inhibitory control in BED and obesity. Based on the evidence of impaired inhibitory control in BED, affected patients might profit from interventions that address impulsive behavior.

## 1. Introduction

Patients suffering from Binge Eating Disorder (BED) show an eating pattern that is characterized by recurrent episodes during which they ingest an unusually large amount of food in a discrete period of time, and experience a subjective loss of control over their eating behavior [1]. As BED patients do not regularly compensate for caloric intake, many patients are overweight or obese [2].

The specific disinhibited eating pattern of BED patients has provoked the assumption that BED might represent a phenotype within the obesity spectrum that is characterized by increased impulsivity [3,4,5]. Impulsivity is understood as a multi-dimensional personality trait with a strong neurobiological basis [4,6]. Theoretical concepts, rooted in personality psychology, have suggested slightly different localizations of impulsivity within the trait hierarchy and different sub-dimensions of impulsivity [7,8]. Most concepts have in common that they differentiate two sub-dimensions of impulsivity: (a) a motivational dimension, closely tied to reward-related processes and (b) a behavioral dimension, related to inhibition. In the present review, we will follow the nomenclature by Dawe and Loxton [9] and term the motivational dimension reward sensitivity, and the behavioral dimension rash-spontaneous impulsiveness. Based on these two dimensions, an impulsive individual would be characterized by persistently seeking out rewards and “acting rashly without consideration of consequences” (p. 345), especially if anticipating a reward [9]. Contemporary theoretical and empirical work has identified a third “impulsigenic trait” that is tied to neuroticism and negative emotionality, hence emphasizing the importance of integrating emotional/affective processes into the framework of impulsivity [10]. Interestingly, many of the early and enhanced theoretical assumptions on the structure of impulsivity have, by now, been supported by identifying underlying neural networks and neurotransmitter signaling, using modern brain imaging techniques [6]. This evidence underpins the strong neurobiological basis of impulsivity.

In their influential work, Dawe and Loxton [9] have proposed how the general concept of impulsivity might be applied to clinical conditions, including disordered eating behavior, and have recently updated this framework [11]. Of note, this update also includes the two original facets of impulsivity and does not include affective/emotional facets. An individual high in reward sensitivity might experience (particular) food as very rewarding and, in a second step, high rash-spontaneous impulsiveness might cause this individual problems in resisting the temptation of this reward. This interplay might lead to the ingestion of food, which might be accompanied by a loss of control over eating, as is seen during binge eating in BED [9]. When Dawe and Loxton suggested this relationship in 2004, there was “less empirical support of this proposal” (p. 347), as the authors put it. This could have been due to the fact that, although binge eating is also seen in other eating disorders (ED), BED first became a formal ED diagnosis in the Diagnostic and Statistical Manual of Mental Disorders (DSM-5) in 2013 [1].

In the meantime, there is evidence suggesting that patients suffering from BED show higher trait impulsivity as compared to healthy normal-weight individuals, but potentially also compared to body mass index (BMI)-matched individuals. For example, in a recent large questionnaire study, BED patients reported significantly more impulsive behaviors than BMI-matched controls [12]. Further support for higher trait impulsivity in BED comes from the co-occurrence of BED with other impulse-control disorders. A recent review and meta-analysis showed a considerable comorbidity between Attention-Deficit/Hyperactivity Disorder (ADHD) and Eating Disorders (ED), including BED [13]. The authors also emphasized that “the risk for the association between ADHD with ED is more than the double that found between obesity and ADHD” (p. 1054) and suggest that one aspect underlying the co-occurrence of ADHD and weight disorders might indeed be binge eating [13]. The comorbidity of ADHD and ED might emerge on the basis of common underlying mechanisms, such as difficulties in reward processing and disinhibition, i.e., a common underlying factor of increased impulsivity. Facets of impulsivity can also be assessed via a range of neuropsychological tasks. The picture concerning this research approach is less clear concerning increased impulsivity in BED patients: In some studies, patients suffering from BED show impaired functioning, as compared to normal-weight controls and to obese individuals without BED, e.g., in abilities associated with executive functioning [4]. However, two recent reviews and meta-analyses found no difference between obese individuals, with and without BED, regarding tasks assessing reward-related decision-making [14] and inhibitory control [15]—although these findings were partly based on a limited number of studies.

The evidence summarized in the preceding paragraph focusses on general impulsivity, using general tasks and stimuli; and this could partly be a reason for the heterogeneity of findings. Kittel et al. [16] differentiated between neuropsychological tasks, using general stimuli vs. disorder-related stimuli (predominantly food), and concluded that BED patients perform more poorly than BMI-matched, as well as normal-weight controls, especially when disorder-related information is involved. Other studies assessed food craving in patients with BED, resembling reward sensitivity concerning food, and found differences in comparison to obese patients without BED, particularly concerning sweets [17,18,19]. A food-related focus in the investigation of impulsivity might allude to processes closer to the core pathology of BED, e.g., as theoretically suggested in the framework of food-related impulsivity by Dawe and Loxton [9], outlined above. In line with this, we have previously performed a systematic review, analyzing experimental and observational studies on food-related impulsivity in patients with BED [5]. We included 51 studies reporting direct behavioral or physiological measures of both reward sensitivity and rash-spontaneous behavior [9]. We were specifically interested in the question of whether BED patients would show increased impulsivity in one or both facets of impulsivity, as compared to obese individuals without BED. Indeed, at that time, evidence supported the view of BED being a phenotype within the obesity spectrum characterized by increased food-related impulsivity. However, most studies had investigated reward-related aspects of impulsivity, and at that time, there were only few experimental studies investigating food-related rash-spontaneous behavior, which predominantly looked at mixed samples of obese individuals, with and without BED. The evidence on this aspect was therefore difficult to interpret. Based on the evidence base at that time, we concluded that (1) obese individuals without BED show increased reward sensitivity as compared to normal-weight individuals, and individuals with BED are characterized by an even greater reward sensitivity, as compared to obese individuals without BED; and (2) that obese individuals with BED also show increased rash-spontaneous behavior, while this was less clear in obese individuals without BED, due to a lack of studies.

Since our review in 2013 when BED was introduced into DSM-5 [1], a growing interest in mechanisms underlying this ED has stipulated research [4,14,15,16]. Here, we provide an update on the evidence on food-related impulsivity in BED. Our review complements other current reviews in the field, by focusing on food-specific processes in BED and by building on the theoretical framework by Dawe and Loxton [9]. We will again differentiate evidence on reward sensitivity vs. rash-spontaneous behavior, but we acknowledge at the same time, emotional/affective processes as important facets of the impulsivity concept. Reviews on the role of emotion regulation in BED have recently been provided by our group [20] and other colleagues [16], and this is also a topic of the work by Dingemans et al., in this issue.

As with our previous review, the present update seeks to summarize evidence on food-related impulsivity in obese individuals, with and without BED, as well as normal-weight individuals. We will address the following main research questions: (1) Is the initial evidence showing increased food-related impulsivity in obese individuals with BED as compared to obese individuals without BED and normal-weight individuals consolidated by novel studies? (2) Is this increased food-related impulsivity equally expressed in the facets of reward sensitivity and rash-spontaneous behavior?

## 2. Materials and Methods

In conducting this updated systematic review, we followed the guidelines of the PRISMA statement (preferred reporting items for systematic reviews and meta-analyses) [21]. As we were conducting an update of our previous systematic review [5], we relied on the same established search strategy, eligibility criteria and study selection procedure.

### 2.1. Search Strategy

We searched the scientific databases, PubMed and PsychInfo, for relevant publications. We restricted our search, for the present update, to articles published since 2012, as earlier publications have been reported in our previous systematic review [5]. We used the identical search terms as within our previous systematic review [5] and again, narrowed the search to titles and abstracts of publications. For the PubMed search, this search term was:

((obesity (MeSH Terms) OR overweight (MeSH Terms) OR obes* (Title/Abstract) OR binge-eating disorder (MeSH Terms) OR “binge eating” (Title/Abstract) OR “binge-eating” (Title/Abstract) OR “BED” (Title/Abstract) OR Hyperphagia (MeSH Terms) OR overeating (Title/Abstract)) AND (food (MeSH Terms) OR food (Title/Abstract) OR eating (MeSH Terms)) AND (impulsive behavior (MeSH Terms) OR impulsiv* (Title/Abstract) OR reward (MeSH Terms) OR disinhibit* (Title/Abstract) OR “loss of control” (Title/Abstract))).

The PsychInfo search term was identical, with the exception of “MeSH Terms”, as they are not defined in PsychInfo. We additionally performed a hand search through potentially relevant articles cross-cited in search results and inspected the reference list of recently published reviews in the field [4,14,15,16].

### 2.2. Eligibility Criteria

Eligibility criteria were based on the PICOS taxonomy, as recommended by the PRISMA statement [21], defining criteria for each of the five PICOS-domains, i.e., *participants (P)*, *interventions (I)*, *comparators (C)*, *outcome (O)* and *study design (S)*.

*Participants:* Studies were eligible if they were conducted with human subjects, across the whole age spectrum. Participants had to be classifiable into one of three groups: (1) overweight/obese participants (BMI > 25 kg/m^2^), in whom a diagnosis of BED was excluded through expert interview or self-report (OB group); (2) overweight/obese participants (BMI > 25 kg/m^2^) in whom a diagnosis of BED was not excluded through expert interview or self-report (OB/BED group); (3) participants with a diagnosis of a full-syndrome or sub-clinical BED, according to expert interview or self-report (BED group). Studies were excluded if they were conducted with participants fulfilling the diagnosis of another eating disorder (esp. anorexia or bulimia nervosa) or a severe mental or neurological disorder (e.g., Prader Willi Syndrome, dementia).

*Interventions (in this case: independent variables):* In order to be included, studies had to use at least one food-related independent variable, e.g., presentation of food pictures, smell or taste of food, food words or food consumption.

*Comparators:* Studies were eligible if a control group was included that consisted of either normal-weight participants (BMI > 19 kg/m^2^ and < 25 kg/m^2^) (NWC) or, in comparison to a BED group, of participants in whom BED has been excluded (Non-BED group).

*Outcome:* In order to be included, studies had to report at least one outcome reflecting one of the facets of impulsivity according to Dawe and Loxton (reward sensitivity and rash-spontaneous behavior) [9].

*Study design:* Experimental and observational studies were included.

## 3. Results

### 3.1. Study Selection

**Figure 1:**
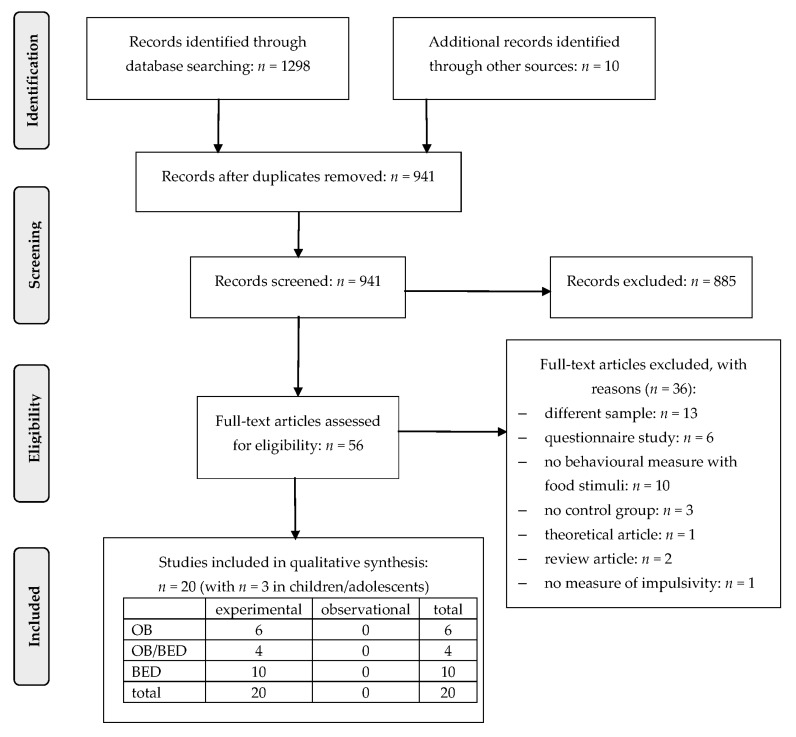
PRISMA (preferred reporting items for systematic reviews and meta-analyses) [21] flow chart for study inclusion. OB: obese sample; OB/BED: mixed sample; BED: sample with Binge Eating Disorder.

We identified 1298 publications through our systematic search and 10 publications through other sources (see Figure 1). Throughout the screening process, 56 publications remained for the full-text analysis. Thirty-six of these publications did not fulfill one or more of the eligibility criteria and were excluded, resulting in 20 studies that were finally analyzed for the present systematic update of the literature.

All of the 20 included studies had an experimental design. Half of the studies investigated BED samples, the remaining predominantly focused on obese samples. Most samples consisted of adults; only three studies investigated children or adolescents. Generally, the included studies were of high methodological variety, reporting data from brain imaging, psychophysiology, neuropsychology or combinations of different measures. The majority of studies controlled for prior food intake and/or hunger levels. Twelve studies had a sole focus on reward sensitivity, five focused on rash-spontaneous behavior and three studies investigated both impulsivity components.

### 3.2. Studies Investigating Food-Related Reward Sensitivity

Most of the twelve studies assessing food-related reward sensitivity investigated adult samples and two were on adolescents [22,23]. Six studies investigated food-related reward sensitivity in BED samples [23,24,25,26,27,28]. Four studies assessed attentional biases for food pictures, in different paradigms, as an indicator of higher food-related reward sensitivity in adults [25,26,28] and in adolescents [23]. All studies in adults found early attentional biases for food pictures in BED participants, as compared to OB participants, while such an early attentional bias was not found in the adolescent population [23]. However, adolescent BED patients showed an attentional bias for food pictures in later processing stages [23], and such a bias in later food processing was also found in adult BED patients by Schag et al. [28]. Leehr et al. [24] assessed facial muscle activity during a valence rating and while the BED group rated the food pictures more positively than both control groups (OB, NWC), there were no group differences found in facial muscle activity. Simon et al. [27] investigated brain responses to a monetary and food incentive delay task in different samples, including obese individuals, with and without BED. No differences in brain activity were found in brain areas that are typically involved in the processing of rewards between the BED patients and non-BED controls [27].

Taking a look at food-related reward sensitivity in OB and OB/BED samples, the evidence from behavioral tasks was mixed: One study asked OB versus NWC participants to rate the palatability of high- and low-caloric food pictures and found no group differences in this palatability rating [29]. Three studies assessed attentional biases for food pictures [30,31,32], using slightly different tasks. One of these studies, with a comparably large sample size (*n* = 319), did not find general group differences between OB and NWC participants, but in a post-hoc analysis, based on self-reported impulsivity, built groups of high-impulsive versus low-impulsive participants [31]. In this post-hoc analysis, high-impulsive OB participants were significantly faster than high-impulsive NWC participants in detecting a high-caloric food item among neutral items, indicating higher food-related reward sensitivity. The second study, using an attention deployment approach, found that OB participants had more difficulty with attentional disengagement from food pictures, as compared to NWC participants [32], while a third study comparing a mixed OB/BED sample with a NWC sample found no group differences in reward sensitivity [30]. This could be due to the heterogeneous sample or due to potentially reduced rewarding properties of food words, as compared to visual material in this study [30]. Schiff et al. [33] assessed a specific facet of reward sensitivity: temporal discounting, a concept expressing the strength of the tendency to choose immediate (potentially smaller) rewards in favor of long-term gains. They found that OB participants showed a stronger tendency to prefer immediate food rewards than NWC participants, while there was no group difference for other rewards. Three studies reported data on brain activity in response to visual food pictures [34,35] or milkshake intake [36]. They provided some evidence for altered activity in brain areas of the reward system in OB and OB/BED participants, as compared to NWC participants, e.g., the anterior cingulate cortex and the ventral striatum of OB and OB/BED participants showed higher activity in response to food pictures; however, in the study by Martens et al. [35], this occurred only in the fasted and not in the satiated state. However, Babbs et al. [36], who provided milkshake intake in the scanner, came to different conclusions, as they predominantly found lower recruitment of the caudate nucleus in the OB group than in the NWC group. When they provided odors, the groups did not differ. Thus, the results from this study were inconclusive and hard to interpret. Of note, the functional magnetic resonance imaging (fMRI) study by Martens et al. [35] is the only one in this review to systematically vary hunger versus satiety. They found differential brain activity patterns, depending on homeostatic state, with more intense reward signaling in the OB/BED group when hungry and decreased reward signaling when satiated. One study investigated electrocortical response to food pictures in OB/BED adolescents versus a NWC group and found no group differences, which might be due to the heterogeneous OB/BED group or due to the adolescent sample [22].

### 3.3. Studies Investigating Food-Related Rash-Spontaneous Behavior

We identified eight studies which had a focus on food-related rash-spontaneous behavior [28,29,30,37,38,39,40,41], one of them investigating an adolescent group (OB/BED vs. NWC) [40]. All of these studies used classical inhibitory control tasks that were adapted using food pictures, most of them requiring a manual response (or the suppression of this response), or assessing oculomotor responses in a modified antisaccade task [28,38]. One study assessed brain activity during the inhibitory control performance [37].

In four out of five studies, BED patients showed difficulties in inhibitory control, as compared to OB and NWC participants, e.g., they had more problems withholding an initiated prepotent reaction [39,41] and suppressing a dominant oculomotor reaction [28,38]. Of note, these deficits were mainly irrespective of stimulus category, which means that they were not enhanced when confronted with food pictures, at least not in early processing stages. The study by Hege et al. [37] is the only one to use a GoNoGo task to assess inhibitory control and to measure brain activity during task performance. The authors found no behavioral differences and no differences in brain activity between BED and OB; however, correlational results indicated a strong association between self-reported impulsivity and recruitment of prefrontal brain areas during response inhibition, especially in BED patients (those with higher trait impulsivity recruited these brain circuits less during response inhibition).

Nederkoorn et al. [40] used the same inhibitory control task as two of the studies in adult BED patients, but in a mixed sample of OB/BED children. They found that the OB/BED group had problems with suppressing reactions towards food pictures, but not to the control pictures, indicating food-specific inhibitory control deficits.

The two studies investigating a mixed OB/BED sample and an OB sample vs. NWC participants, respectively, found no group differences in rash-spontaneous behavior [29,30]. In the case of the study on OB/BED, this could be due to the heterogeneous sample or due to potentially reduced rewarding properties of food words, as compared to visual material [42]. A methodological strength of the study by Mühlberg et al. [29] is that the authors used individually preferred food items for the inhibitory control task, which supposedly increases task relevance and difficulty; however, this did not result in group differences.

## 4. Discussion

Since our initial systematic review on food-related impulsivity in BED and obesity [5], there has been considerable research activity in the field, which is reflected in a number of experimental studies with a high methodological quality. In the present review, we provided an update and integrated evidence from twenty experimental studies that have been conducted in the meantime in this field. Half of the studies investigated BED samples, the remaining have predominantly focused on obese samples. Compared to the studies included in our initial review [5], most of the studies on obese samples now control for a comorbid ED or exclude individuals with BED, which represents a methodological improvement in the field.

### 4.1. Summary and Interpretation of Results

Summarizing the current findings, there is congruent evidence for increased food-specific reward sensitivity in BED patients, as compared to BMI-matched controls and—depending on the study design—also normal-weight controls. Some studies also found increased food-specific reward sensitivity in OB samples, although the evidence was more mixed for this group. Regarding rash-spontaneous behavior, the majority of studies investigated BED samples and, except for one study, all of them found increased rash-spontaneous behavior in BED, that is, decreased inhibitory control. However, the picture is mixed with regard to whether these deficits seen in BED are rather general or food-specific. OB control samples in these studies showed lower inhibitory control deficits than BED patients, or were comparable to NWC controls, indicating largely intact inhibitory control. Only a few studies investigated heterogeneous samples of OB/BED individuals, and they showed inconclusive findings, partly no differences to the NWC control group, and this might partly be due to the mixed sample.

Taking a look beyond our categorization of weight groups and diagnostic groups, some studies which have not found group differences have reported evidence for trait impulsivity as a dimensional underlying factor of task performance, as revealed by correlational analyses [22,29,31,37]. For example, in the study by Mühlberg et al. [29], the ability to withhold reactions to food was strongly influenced by trait impulsivity and less by BMI.

**Figure 2:**
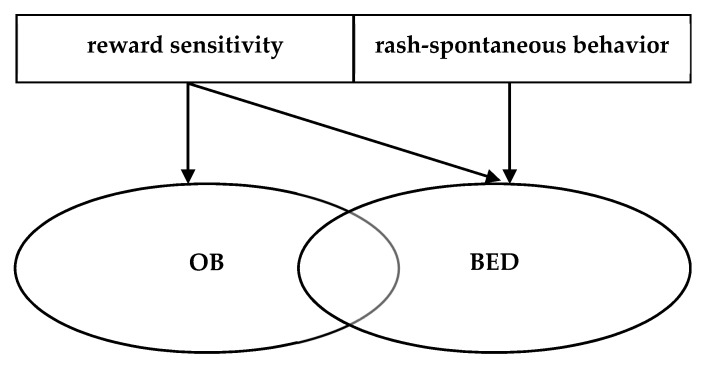
Model of impulsivity deficits in obese patients with and without BED, adapted from Schag et al., 2013 [5]. OB: patients with obesity; BED: patients with Binge Eating Disorder.

Coming back to our initial research questions for the present update of the literature, the evidence partly supports question (1): obese individuals with BED show increased impulsivity as compared to obese individuals without BED and normal-weight individuals, at least with regard to rash-spontaneous behavior. However, this impulsivity might not be exclusively food-specific, but also expressed in general terms. This conclusion also answers research question (2) on the facets of impulsivity, which is summarized in Figure 2: It hence seems like obese individuals, with and without BED, do not necessarily differ that much in the dimension of reward sensitivity, although, as outlined above, the evidence for OB samples remains mixed. We cautiously conclude from the current evidence that both groups might experience food as more rewarding than NWC individuals, but OB individuals seem to have less problems inhibiting their behavior towards this reward, while BED patients show rash-spontaneous behavior towards food and also under general conditions requiring inhibitory control.

These conclusions are largely in line with the evidence base which we synthesized in our previous review [5]. Back then, we concluded that food-related reward sensitivity was also increased in OB individuals, but to a stronger extent in BED individuals. Based on the additional evidence, this still holds true; however, the evidence is more mixed, leaving rash-spontaneous behavior as clearer and major discrimination characteristic of obese individuals, with and without BED.

### 4.2. Methodological Considerations

Most of the studies that are summarized in the present review have investigated well-defined samples either classifying for an OB or a BED group, there were only few studies with a heterogeneous OB/BED sample where an ED was not assessed or excluded in obese participants. This is a major improvement for the interpretation of results and for the investigation of BED as a potential distinct subgroup within the obesity spectrum.

A further methodological strength of the summarized evidence is the control or assessment of previous food intake—the majority of studies reported that participants were assessed after standardized fasting times or standardized preloads. The study by Martens et al. [35] demonstrated that homeostatic state is an important variable influencing food cue processing—in this case, reward sensitivity was modulated by hunger vs. satiety in the participants. However, this study was the only one among the included studies which systematically varied homeostatic state (i.e., hunger vs. satiety).

A major source of heterogeneity in the field was the paradigms/methods and the food stimulus materials used. On one side, consistent evidence from different methodological approaches certainly increases trust in the validity of findings. However, especially the heterogeneity of food items/pictures used in the different studies poses problems in the comparability and reproducibility of findings as they might have a major impact on group differences [42]—specific foods might be more rewarding to some participants than for others. Table 1 shows that studies used material with different caloric contents, which might also impact the findings. One of the reviewed studies used individually preferred food items for the inhibitory control task [29], which might increase task relevance and ecological validity; however, this did not result in group differences.

A broader methodological limitation that affects experimental research on the regulation of eating behavior in general has been pointed out in a recent review by Berner et al. [43]: Experimental studies are conducted under laboratory conditions and mostly do not include any consummatory behavior. The behavior and reactions shown under these laboratory circumstances certainly differ from those under natural eating conditions and during dysfunctional eating (i.e., binge eating). In the present review, there was only one study included which assessed effects of real food ingestion (a milkshake) [36]. In our previous review, we reported on a range of observational studies which entailed real food consumption [5]; however, no observational study that complied with our inclusion criteria for the present review has been published in the meantime.

### 4.3. Conclusions and Future Directions for Research and Therapy

In the present review, we provided a systematic update of the evidence on food-related impulsivity in BED and obesity that has been published since our initial systematic review on this topic [5]. We have synthesized findings from twenty experimental studies that have been published and have come to the conclusion that BED represents a distinct phenotype within the obesity spectrum that is characterized by increased rash-spontaneous behavior in general and specifically towards food, while food-specific reward sensitivity is also increased in obese individuals without BED, but potentially to a lesser degree.

Taking a closer look at the evidence for this discrimination characteristic, it is important to note that rash-spontaneous behavior–or inhibitory control, as it is mostly assessed by the present experimental study paradigms–is not a unidimensional concept, but is built of sub-domains itself, so classical experimental tasks and paradigms also assess slightly different aspects of inhibitory control [11,44]. Evidence also suggests that early versus late inhibitory processes might be differentially affected in BED. A major next step for research therefore entails the investigation and disentanglement of the roles of these sub-domains and temporal components of inhibitory control in BED and obesity. Preliminary evidence also points to a relationship between inhibitory control and genetic outfit [38,45]. Taking into account that impulsivity has a strong neurobiological basis [6], this line of research is also promising for a deeper understanding of dysfunctional (eating) behavior across the obesity spectrum.

Another construct that is currently much discussed in BED and obesity research, is the concept of food addiction [46,47,48]. Food addiction has a high overlap with BED and obesity [47] and is positively related to increased reward sensitivity and inhibitory control [49,50,51,52,53]. Thus, besides the differentiation of obese patients, with and without BED, further research should also be established to disentangle the concepts and mechanisms of BED, obesity and food addiction.

Concerning future treatment options, the study by Jensen and Kirwan [34] integrated a very interesting participant group–individuals who have successfully maintained weight loss. They showed increased activity in brain areas responsible for inhibitory control when confronted with food pictures, and this suggests a pivotal role of inhibitory control capacities for success of weight loss treatments and the regulation of eating behavior. Based on the evidence for impaired inhibitory control in BED, affected patients might profit from interventions that address impulsive behavior, including specific training approaches, psychotherapy or non-invasive brain stimulation [54,55,56,57].

## Figures and Tables

**Table 1:** Experimental studies investigating food-related impulsivity.

Study	Sample ^1^	BMI (M ± SD) in Adults/BMI-SDS in Adolescents	*n*	Stimuli	Task/Outcome ^2^	Results ^3^
Babbs et al. (2013) (Study 1) [36]	OW/OB	31.2 ± 1.4	13	Odor and ingestion of chocolate milkshake vs. tasteless control solution	- Pleasantness ratings of odors and milkshake - Brain response to odors and milkshake (fMRI) after a 2 h fast	Pleasantness ratings: OW/OB = NWC Brain activity: - To odors: OW/OB = NWC - To ingestion: NWC > OW/OB to milkshake vs. control solution in caudate nucleus and dorsal striatum; OW/OB > NWC to milkshake vs. control solution in ventral putamen
	NWC	21.9 ± 0.5	12			
Bongers et al. (2015) [31]	OB	38.2 ± 6.2	185	Pictures of neutral items vs. high-caloric foods vs. low-caloric foods	Attentional bias for high- and low-caloric foods in a visual search task after a 2 h fast	OB = NWC Group differences only after integrating self-reported trait impulsivity: HI OB > HI NWC for high-caloric food vs. neutral items
	NWC	22.4 ± 1.6	134			
Carters et al. (2015) [32]	OB	35.6 ± 4.8	29	Pictures of neutral items vs. high-caloric foods	Attentional disengagement from food, as assessed by RT, in inhibition of return task, after a 1 h fast	OB > NWC in RT for food pictures concerning attentional disengagement
	NWC	22.1 ± 1.6	35			
Hege et al. (2015) [37]	BED	34.0 ± 5.6	17	Pictures of neutral items vs. high-caloric foods	Food-related GoNoGo task 1. RT and accuracy 2. Magnetic brain activity (MEG) after a standardized breakfast	1. BED = OB in RT and accuracy of a food-related GoNoGo task 2. BED = OB in MEG activity; increased self-reported attentional impulsiveness was related to hypoactivity in the prefrontal control network during NoGo trials, irrespective of stimulus category
	OB	36.5 ± 4.9	17			
Hofmann et al. (2015) [22]	OB/BED	2.5 ± 0.3	34	Pictures of neutral items vs. food items	Electrocortical response (ERP: P100 and P300) in a passive viewing task after a 3 h fast	OB/BED = NWC in an electrocortical response
	NWC	0 ± 0.9	24			
Jensen & Kirwan (2015) [34]	OW/OB	31.4 ± 1.3	11	Pictures of high-energy foods vs. low-energy foods	Brain response (fMRI) in a passive viewing task after a 4 h fast	Brain activity: SWL > NWC, OW/OB to high-energy food pictures in DLPFC and superior frontal gyrus OW/OB > SWL to food pictures in ACC and vmPFC OW/OB > SWL, NWC to food pictures in VS
	SWL	23.0 ± 2.6	11			
	NWC	22.0 ± 2.1	12			
Leehr et al. (2016) [38]	BED	34.4 ± 5.5	21	Pictures of neutral items vs. high-caloric foods	Failure to inhibit saccades towards pictures in a food-related antisaccade task	BED > OB, NWC in saccade errors, irrespective of stimulus category
	OB	33.2 ± 4.2	23			
	NWC	22.3 ± 1.7	25			
Leehr et al. (2016) [24]	BED	34.7 ± 5.1	16	Pictures of neutral items vs. high-caloric food items	1. Valence rating (wanting and liking) 2. Facial muscle activity, as assessed by EMG after a standardized breakfast	1. BED > OB, NWC concerning valence of stimuli 2. BED = OB = NWC in EMG
	OB	33.4 ± 4.5	23			
	NWC	22.1 ± 1.6	22			
Loeber et al. (2012) [30]	OB/BED	38.8 ± 6.3	20	1. Food-related and neutral words 2. Pictures of neutral items vs. foods items	1. RT and accuracy in a food-related GoNoGo task 2. Attentional bias to food pictures (RT) in a food-related Visual Dot Probe task after a 3 h fast	1. OB/BED = NWC in RT and accuracy of a food-related GoNoGo task 2. OB/BED = NWC in RT of a food-related Visual Dot Probe task
	NWC	22.6 ± 1.1	20			
Manasse et al. (2016) [39]	BED	35.2 ± 7.7	25	Pictures of neutral items vs. pleasant items vs. highly palatable foods	SSRT in a food-related Stop Signal Task	BED > OB in SSRT of a food-related Stop Signal task, irrespective of stimulus category
	OB	36.7 ± 5.54	65			
Martens et al. (2013) [35]	OB/BED	28.1 ± 0.3	20	Pictures of neutral items vs. food items	Brain response (fMRI) in a passive viewing task 1. In a fasted state 2. after a standardized breakfast	Brain activity: 1. OB/BED > NWC to food pictures in the ACC 2. OB/BED < NWC to food pictures in the ACC and PFC
	NWC	22.7 ± 0.2	20			
Mühlberg et al. (2016) [29]	OB	34.3 ± 2.3	26	Pictures of neutral items vs. palatable high-caloric foods vs. palatable low-caloric foods	1. Palatability rating in a Food Picture Rating Task 2. RT, accuracy and SSRT in a food-related Stop Signal Task after a 1 h fast	1. OB = NWC in a food palatability rating 2. OB = NWC in RT, accuracy and SSRT in a food-related Stop Signal Task
	NWC	21.8 ± 1.9	30			
Nederkoorn et al. (2012) [40]	OB/BED	21.1 ± 2.7	14	Pictures of attractive toys vs. palatable food items	SSRT in a food-related Stop Signal Task	OB/BED > NWC in SSRT for food pictures in a food-related Stop Signal Task
	NWC	16.1 ± 1.5	75			
Schag et al. (2013) [28]	BED	35.4 ± 5.6	25	Pictures of neutral items vs. food items	1. Attentional bias for food pictures in an ET free exploration task, as assessed by initial fixation position and total gaze duration 2. Directional errors in a food-specific antisaccade task after a standardized breakfast	1. BED = OB = NWC in initial fixation duration BED > OB, NWC in total gaze duration on food pictures 2. BED > OB, NWC in directional errors in the first saccade irrespective of stimulus category BED > OB, NWC in directional errors in the second saccade towards food pictures
	OB	35.4 ± 5.4	26			
	NWC	22.5 ± 1.6	25			
Schiff et al. (2016) [33]	OB	36.2 ± 5.7	23	Preferred food vs. preferred discount voucher vs. money	Temporal discounting functions for the different rewards in a temporal discounting task	OB > NWC for future food reward (i.e., preference of immediate food reward) OB = NWC for voucher and money rewards
	NWC	22.4 ± 2.2	23			
Schmidt et al. (2016) [23]	BED	1.77 ± 0.95	25	Pictures of neutral items vs. food items	1. Attentional bias for food pictures in an ET free exploration task, as assessed by initial fixation position and total gaze duration 2. Attentional bias for food pictures in an ET visual search task, as assessed by RT for detection of food among non-food distractors–RT for detection of non-food among food distractors after a 1 h fast	1. BED = OB in initial fixation duration BED > OB in total gaze duration on food pictures 2. BED > OB in RT for attentional bias towards food pictures
	OB	1.77 ± 0.82	25			
Schmitz et al. (2014) [26]	BED	34.7 ± 5.1	27	Pictures of neutral items vs. high-caloric food items	RT in a Dot Probe Task for 1. Congruent trials (stimulus engagement) 2. Incongruent trials (stimulus disengagement)	1. BED > OB in stimulus engagement for food stimuli 2. BED = OB in stimulus disengagement
	OB	32.4 ± 6.4	33			
Schmitz et al. (2015) [25]	BED	35.7 ± 6.5	25	Neutral words vs. food words	Attentional bias for food, as assessed by accuracy in a Rapid Serial Visual Presentation Paradigm for 1. Stimulus engagement 2. Stimulus disengagement after a 2 h fast	1. BED > OW/OB in stimulus engagement for food stimuli 2. BED = OW/OB in stimulus disengagement
	OW/OB	32.9 ± 6.0	30			
Simon et al. (2016) [27]	BED	32.6 ± 4.6	27	Money and snack points exchangeable for real money, snacks, beverages and fruits immediately after the fMRI measurement	Brain response (fMRI) in a Monetary and Food Incentive Delay Task after a standardized breakfast	Brain activity during receipt of high vs. no food reward: BED > OB in posterior cingulum, hippocampus and dorsal medial cingulum
	OB	34.0 ± 4.5	28			
	BN	21.3 ± 3.0	29			
	NWC	21.9 ± 1.9	27			
Svaldi et al. (2014) [41]	BED	35.0 ± 5.1	31	Pictures of neutral items vs. appetizing food items	Food-related Stop Signal Task 1. SSRT 2. Accuracy	1. BED > OB in SSRT, irrespective of stimulus category 2. BED < OB in accuracy concerning food stimuli
	OB	33.0 ± 6.0	29			

^1^ BED: Binge Eating Disorder; sub-BED: subthreshold Binge Eating Disorder; BMI: Body Mass Index; BMI-SDS: Body Mass Index Standard Deviation Score; BN: Bulimia Nervosa; HI: High impulsiveness; LI: low impulsiveness; NWC: normal weight control sample; OB: Obese; OB/BED: mixed sample (BED not assessed or excluded); OW/OB: Overweight and Obese; OW: Overweight; SWL: successful weight losers; ^2^ EMG: electromyography; ERP: event-related potential; ET: eye tracking; fMRI: functional magnetic resonance Imaging; MEG: Magnetoencephalography; RT: reaction time; SSRT: stop signal reaction time; ^3^ ACC: anterior cingulate cortex; PFC: prefrontal cortex; DLPFC: dorsolateral PFC; vmPFC: ventromedial PFC; VS: ventral striatum.
